# 胸腺恶性肿瘤术后静脉血栓栓塞症的影响因素分析

**DOI:** 10.3779/j.issn.1009-3419.2021.101.22

**Published:** 2021-07-20

**Authors:** 兴国 杨, 辉 李, 磊 于, 涛 于, 飞 李, 振 余, 葆勋 张, 鑫 杜

**Affiliations:** 1 100730 北京，首都医科大学附属北京同仁医院胸外科 Department of Thoracic Surgery, Beijing Tongren Hospital, Capital Medical University, Beijing 100730, China; 2 100020 北京，首都医科大学附属北京朝阳医院胸外科 Department of Thoracic Surgery, Beijing Chaoyang Hospital, Capital Medical University, Beijing 100020, China

**Keywords:** 胸腺恶性肿瘤, 危险因素, 静脉血栓栓塞症, Caprini评分, Thymic malignancies, Risk factors, Venous thromboembolism, Caprini score

## Abstract

**背景与目的:**

胸外科术后静脉血栓栓塞症（venous thromboembolism, VTE）的发生率高，本研究旨在分析胸腺恶性肿瘤术后VTE的发生率及相关危险因素。

**方法:**

本研究为单中心研究。收集2017年12月-2021年2月在首都医科大学附属北京同仁医院胸外科接受胸腺恶性肿瘤切除手术治疗的患者资料。除术前常规检查以外，所有患者于手术前后行下肢多普勒超声以明确有无下肢深静脉血栓（deep venous thrombosis, DVT）。患者术前术后均未接受任何形式抗凝治疗。全部患者接受改良Caprini风险评估。根据术后是否发生VTE将患者分为VTE组和对照组，比较两组间的临床资料，统计术后VTE的发生时间、可能的高危因素等。

**结果:**

共纳入169例接受胸腺恶性肿瘤切除手术的患者，男性94例，女性75例，年龄范围：22岁-76岁。本研究共95例患者选择胸腔镜手术，74例选择正中劈胸骨手术。全组共21例发生VTE，VTE的总发生率为12.4%。诊断VTE的中位时间为术后4 d（2 d-15 d）。根据改良Caprini评分分组，低危组（≤4分)发生率为0%（0/7），中危组（5分-8分）为7.0%（8/115），高危组（≥9分）为27.7%（13/47）（*Z*=1.670, *P*=0.008）。单因素分析结果显示VTE组年龄、手术方式、手术时间、留置中心静脉导管、术后卧床时间超过72 h均与对照组有显著差异（*P* < 0.05）。多因素分析结果显示年龄≥60岁、手术方式、手术时长是胸腺恶性肿瘤术后发生VTE的独立危险因素。

**结论:**

年龄≥60岁、手术方式、手术时长是胸腺恶性肿瘤患者术后发生VTE的独立危险因素，改良Caprini评分可有效筛选高危患者。

## Abstract (en)

**Background and objective:**

The previous study has indicated that the incidence of venous thromboembolism (VTE) after thoracic surgery is high. The purpose of this study was to analyze the incidence and risk factors of postoperative VTE in thymic malignancy patients.

**Methods:**

This was a single-center study. Patients undergoing resection for thymic malignancy between December 2017 and February 2021 in Department of Thoracic Surgery, Beijing Tongren Hospital Affiliated to Capital Medical University were enrolled in this study. In addition to the routine examination, all patients were screened for deep venous thrombosis (DVT) by using noninvasive duplex lower extremity ultrasound before and after surgery. Patients did not receive any prophylactic anticoagulant therapy before and after surgery. All patients received modified caprini risk assessment. According to whether VTE occurred after operation, patients were divided into VTE group and control group. The clinical data of the two groups were compared. The occurrence time and possible high risk factors of VTE after operation were analyzed.

**Results:**

A total of 169 patients with thymic malignant tumor were enrolled, including 94 males and 75 females, aging from 22 to 76 years. A total of 95 patients underwent thoracoscopic surgery and 74 patients underwent median sternotomy. The total incidence of VTE was 12.4%. The median time for diagnosis of VTE was 4 days (2 days-15 days) after operation. According to the modified caprini score, the incidence of VTE in low risk patients (Caprini score≤4 points), moderate risk patients (Caprini score 5 to 8 points) and high risk patients (Caprini score≥9 points) were 0% (0/7), 7.0% (8/115) and 27.7% (13/47), respectively (*Z*=1.670, *P*=0.008). Univariate analysis showed that there were significant differences between VTE group and control group in age, operation method, operation time, indwelling central venous catheter, postoperative bed rest time more than 72 hours (*P* < 0.05). Multivariate analysis showed that over 60 years old, operation method and operation time were independent risk factors for VTE after resection for thymic malignancies.

**Conclusion:**

Over 60 years old, operation method and operation time are independent risk factors for VTE. Modified caprini assessment can effectively screen high-risk patients.

静脉血栓栓塞症（venous thromboembolism, VTE）包括肺栓塞（pulmonary thromboembolism, PE）和深静脉血栓形成（deep vein thrombosis, DVT），是恶性肿瘤患者和外科手术术后常见的并发症之一，VTE是医院意外死亡的重要原因之一。在一项关于恶性肿瘤患者血栓预防的研究^[1]^中，未行肿瘤治疗的患者其DVT的平均发生率为29%。研究^[2, 3]^表明，胸外科手术后VTE发生率较高，然而尚无研究描述胸腺恶性肿瘤切除术后VTE的发生率。为评估胸腺恶性肿瘤切除术后VTE的发生率和发生特点，并确定发生VTE的高风险患者，我们进行了这项单中心研究。

## 1 资料与方法

### 1.1 研究对象选择

本研究为单中心研究，收集2017年12月-2021年2月在首都医科大学附属北京同仁医院胸外科接受胸腺恶性肿瘤手术治疗，且满足本研究入组标准的患者资料。纳入标准：（①诊断胸腺恶性肿瘤；②接受胸腺恶性肿瘤切除手术治疗，术式选择胸腔镜或者正中劈胸骨；③患者年龄≥18岁。排除标准：①缺少临床资料；②患者进行一次以上术后手术干预；③术前检查确诊VTE；④术前、术后接受任何形式抗凝治疗。所有入组患者观察术后住院期间VTE发生情况。

### 1.2 研究方法

本研究所有的患者在术前均通过双功能多普勒超声检查确定的患者未有任何DVT。手术后行下肢多普勒超声以明确有无DVT。如患者术后出现新发DVT，或出现不明原因呼吸困难、咳血、胸痛等症状高度怀疑PE时，则追加行计算机断层扫描肺血管造影（computed tomography angiography, CTPA）除外有无新发PE。所有患者采用波士顿医学中心的改良Caprini评分进行VTE风险评估^[4]^，将手术患者分为低危（≤4分）、中危（5分-8分）和高危（≥9分）三组。

### 1.3 观察指标

收集患者病例的详细信息，包括年龄、性别、体质量指数（body mass index, BMI）、合并症（高血压、糖尿病和冠状动脉粥样硬化性心脏病等）、是否合并自身免疫疾病（重症肌无力等）、手术过程相关信息（手术方法、切除范围、手术时长和失血量等）和肿瘤病理资料。

### 1.4 诊断依据及标准

胸腺恶性肿瘤的诊断以肿瘤病理学结果为依据，肿瘤的分期参照2014年国际肺癌协会（International Association for the Study of Lung Cancer, IASLC）/胸腺肿瘤协作组（International Thymic Malignancy Interest Group, ITMIG）提出的第八版胸腺恶性肿瘤原发灶-淋巴结-转移（tumor-node-metastasis, TNM）分期；DVT经下肢彩色多普勒超声检查诊断，PE经CT肺动脉造影诊断。术后VTE事件定义为术前明确无VTE，术后经下肢多普勒超声诊断为新发DVT（包括肌间静脉血栓）或经CTPA诊断为PE。

### 1.5 统计学处理

本研究所有数据均采用SPSS 26.0统计软件进行分析。计量资料使用均数±标准差（Mean±SD）的形式体现。采用Student *t*检验比较两组连续变量的平均值。分类数据显示为频率分布（*n*）和百分比（%），通过*Mann-Whitney U*检验、χ^2^检验或*Fisher*精确检验分析分类变量，*P* < 0.05表示具有统计学差异。

## 2 结果

### 2.1 一般资料分析

本研究共入选169例胸腺恶性肿瘤患者，其中男性94例，女性75例，患者的年龄为22岁-76岁，中位年龄50岁。入组患者涵盖多个类型的自身免疫疾病，包括重症肌无力76例、多发性肌炎1例、皮肌炎1例、系统性红斑狼疮1例；合并基础病包括高血压47例、糖尿病28例、冠状动脉粥样硬化性心脏病15例、慢性阻塞性肺病64例等。入组患者术后诊断VTE 21例（VTE组），其中DVT 20例，PE 1例；未发生VTE 148例（对照组）。DVT包括肌间静脉、胫静脉、腘静脉、股总静脉，发生在下肢单侧共15例，双侧5例。其中术后5 d内确诊者占76%（16/21），确诊VTE的中位时间为术后4 d（2 d-15 d）。VTE组年龄明显大于对照组（60.6±6.7 *vs* 50.4±10.2, *P* < 0.001）；在性别、BMI、合并免疫疾病、合并基础病等方面，VTE组与对照组无统计学差异（*P* > 0.05）（表 1）。

**表 1:** 术后VTE组和无VTE组的手术患者临床资料比较 Comparison of clinical data between VTE group and non-VTE group

Item	VTE (*n*=21)	Non-VTE (*n*=148)	χ^2^/*t*/*Z*	*P*
Age (yr)			4.429	< 0. 001
< 60	7	112		
≥60	14	36		
Gender			1.583	0.208
Male	9	85		
Female	12	63		
BMI≥25 kg/cm^2^	8	61	0.074	0.785
Autoimmune disease			0.721	0.396
Yes	8	71		
No	13	77		
Comorbidities				
Hypertension	7	40	0.364	0.546
Diabetes	3	25	0.000	1.000
CHD	4	11	1.800	0.180
COPD	10	54	0.969	0.325
Surgical procedure			7.444	0.006
VATS	6	89		
Median sternotomy	15	59		
Bleeding (Mean±SD, mL)	256.7±258.8	175.3±214.5	1.585	0.115
CVC	13	54	4.966	0.026
Length of surgery (Mean±SD, min)	226.2±77.3	171.0±45.5	4.694	< 0.001
Confined to bed (> 72 h)	11	38	6.371	0.012
Pathological type			0.966	0.326
Thymoma (*n*=128)	15	113		
Thymic carcinoma (*n*=33)	6	27		
Pathological staging (Thymoma+Thymic carcinoma)			1.592	0.207
Ⅰ+Ⅱ (*n*=82)	8	74		
Ⅲ+Ⅳ (*n*=79)	13	66		
Caprini score			1.670	0.008
Low risk (0-4)	0	7		
Moderate risk (5-8)	8	107		
High risk (≥9)	13	34		
VTE: venous thromboembolism; BMI: body mass index; CHD: coronary heart disease; COPD: chronic obstructive pulmonary disease; CVC: central venous catheter.				

### 2.2 手术相关因素分析

本组研究手术方式包括经胸胸腔镜手术95例（56%），正中劈胸骨开胸手术74例（44%）；其中选择胸腔镜手术，经右胸83例，经左胸12例。由于肿瘤局部侵犯，对于受侵的局部组织也一并切除，其中肺楔形切除18例，心包部分切除8例。开胸手术患者的术后VTE发生率明显高于胸腔镜手术患者，有显著统计学差异（*P*=0.006）。VTE组手术时间长于对照组，且有统计学差异（*P* < 0.001）（表 1）。

### 2.3 病理相关因素分析

病理结果显示恶性肿瘤包括胸腺瘤128例、胸腺鳞状细胞癌33例、淋巴上皮瘤样癌1例、胸腺类癌1例、淋巴瘤4例、胚胎癌1例以及无性细胞瘤1例。胸腺瘤按世界卫生组织（World Health Organization, WHO）（2004）组织学分型标准进行分型，其中A型6例，AB型22例，B1型13例，B2型42例，B3型18例，B1/B2混合型3例，B2/B3型24例。胸腺瘤及胸腺癌根据TNM分期：Ⅰ期-Ⅱ期82例，Ⅲ期-Ⅳ期79例。病理相关因素显示，病理类型、病理分期在VTE组与非VTE组间无统计学差异（*P* > 0.05）（表 1）。

### 2.4 Caprini评分结果

根据Caprini评分，低风险组7例中无术后VTE发生，发生率为0%；中风险组115例中，8例发生术后VTE，发生率为7.0%，高风险组47例中，13例发生术后VTE，发生率为27.7%。随着Caprini评分的升高，患者发生术后VTE事件的几率明显升高，有显著统计学差异（*P*=0.008）（表 1）。

### 2.5 术后发生VTE影响因素分析

单因素分析结果显示：患者年龄、手术方式、手术时间、留置中心静脉导管、术后卧床时间超过72 h、Caprini评分分组是胸腺恶性肿瘤术后发生VTE的影响因素（*P* < 0.05），而患者性别、BMI、合并重症肌无力、高血压、冠状动脉粥样硬化性心脏病、糖尿病、术中出血量等不是胸腺恶性肿瘤术后发生VTE的危险因素（*P* > 0.05）（表 1）。对于以上分析得到的显著相关性因素进行多因素分析，分析结果显示年龄≥60岁、手术方式、手术时长是患者术后VTE发生的独立危险因素（表 2）。

**表 2:** 胸腺恶性肿瘤术后多因素分析 Multivariate analysis of postoperative complications of thymic malignancies

Item	B	SEM	Wald	*P*	OR (95%CI)
Surgical procedure	1.615	0.808	3.990	0.046	5.027 (1.031-24.519)
Length of surgery	0.012	0.006	4.796	0.029	1.013 (1.001-1.024)
Caprini score	0.559	0.719	0.604	0.437	1.748 (0.427-7.152)
CVC	1.154	0.781	2.182	0.140	0.315 (0.068-1.458)
Confined to bed (> 72 h)	0.356	0.608	0.342	0.559	1.427 (0.433-4.700)
Age≥60 yr	1.644	0.695	5.591	0.018	5.177 (1.325-20.227)

## 3 讨论

胸腺恶性肿瘤主要包括恶性胸腺瘤和胸腺癌，是发生于纵隔最常见的恶性肿瘤，治疗的主要方法是手术治疗^[5, 6]^。目前，国内外已有众多针对外科术后及恶性肿瘤等VTE防治的共识和指南，如美国胸科医师协会（The American College of Chest Physicians, ACCP）、美国国立综合癌症网、英国国家卫生与临床优化研究所等均颁布了VTE防治相关指南^[7-11]^。在一项单中心前瞻性队列研究中，Song等^[12]^发现胸外科手术后未行预防血栓治疗，其VTE总体发生率为13.9%，其中胸部恶性肿瘤患者术后VTE发生率是17.9%，几乎为良性疾病患者的2倍，肺癌和食管癌术后VTE发生率分别为15.9%和27.3%，高于总体发生率。胸外科恶性肿瘤术后VTE发生率较高，但针对胸部恶性肿瘤围术期VTE预防的比例在我国仍比较低，主要原因为多数中国胸外科医生对VTE的认识和关注不足^[13]^。研究证实胸腺恶性肿瘤是发生VTE的高危因素，在我们先前的研究中胸腺良性疾病术后VTE发生率为4.6%，恶性肿瘤发病率为14.5%^[14]^。为明确胸腺恶性肿瘤患者术后发生VTE的相关危险因素，我们开展了这项单中心研究。

目前国内外对于胸外科恶性肿瘤术后VTE发生的研究主要集中于肺癌及食管癌手术，很少针对纵隔肿瘤围术期VTE进行研究^[14, 15]^。然而，纵隔肿瘤在胸部肿瘤中占有很大的比重，胸腺恶性肿瘤手术在纵隔肿瘤中又占有很高的比重。与其他肿瘤不同，胸腺瘤常合并各种自身免疫疾病，如多发性肌炎、类风湿性关节炎、硬皮病、系统性红斑狼疮、重症肌无力等，其中重症肌无力发生率最高，占20.5%-40%。Ramagopalan等^[16]^的研究发现，住院患者合并自身免疫疾病，会增加由免疫介导的血栓风险，对于此类患者可以考虑血栓预防治疗。本研究中共有79例患者合并自身免疫疾病，但是VTE组和对照组相比未显示统计学差异。我们认为这可能与研究对象的选择有关，本研究入组患者合并的自身免疫病主要是重症肌无力，并不会造成全身急性炎症发作而介导的血栓形成。

我们的初步研究发现，手术方式、手术时长在VTE组与对照组间有统计学差异。胸外科开胸手术多数是侧位经肋间进胸，比如肺叶切除或食管切除，而对于胸腺肿瘤手术，开胸手术常选择胸骨正中劈开术式。我们发现选择胸骨正中劈开术式术后VTE发生率为20.3%（15/74），远高于胸腔镜手术后VTE的发生率[6.3%（6/95）]，与传统侧开胸术后VTE发生率接近^[12]^。开胸手术相比于胸腔镜手术创伤大，术后疼痛导致卧床时间更长、术后恢复慢导致住院时间延长或许是VTE发生率增高的原因。另外，手术长时间的制动，及出血量增多导致的凝血平衡的破坏、血流动力学的改变均会增加VTE的发生率^[17]^。

对于胸腺恶性肿瘤患者，筛选出VTE高危风险者，做好积极的围手术期预防可以降低VTE的发生率。现有的VTE风险评估模型有多种，包括Caprini风险评估量表、Rogers评分量表、Padua评分量表和Khorana评分量表等^[4, 18-20]^。美国胸科医师学会在非骨科外科患者VTE预防临床指南中推荐使用Caprini和Rogers评估量表^[10]^。2018年发布的《胸部恶性肿瘤围术期静脉血栓栓塞症预防中国专家共识》推荐使用改良Caprini风险评估模型对胸部恶性肿瘤手术患者进行VTE风险评估^[21]^。本研究入组的胸腺肿瘤切除患者均采用了改良Caprini风险评估模型进行评分，我们发现大部分胸腺恶性肿瘤患者为中高风险，达到95.9%。随着Caprini评分的升高，患者发生术后VTE事件的几率明显升高。对于胸腺恶性肿瘤的患者，尤其是Caprini评分高危风险者，应给予密切关注，提供适当的血栓形成预防措施，从而减少围手术期VTE的发生率。评估应该动态进行，在患者入院和术后都要进行单独评估。另外，如果病情发生重大变化或治疗方案发生改变等，还应进行再次评估。

本研究的优势是入组患者术前术后均未接受抗凝治疗，因此，研究结果很好地反映了VTE真实发生率的自然情况。本研究的局限性：①资料是回顾性的，之前未考虑的其他混杂因素可能影响数据；②我们对入组患者住院期间VTE的发生进行调研，但是患者出院后无长期随访，因此并未探讨患者VTE在出院期间是否也有很高的发生率；③由于CTPA未在所有术后患者中检查，本项研究中可能存在部分无症状PE漏诊的状况。

综上，VTE是胸腺恶性肿瘤患者术后比较常见并发症，年龄、手术方式、手术时长、留置中心静脉导管、术后卧床时间超过72 h是胸腺恶性肿瘤术后VTE的危险因素，年龄≥60岁、手术方式、手术时长是其独立危险因素。改良的Caprini风险评估模型可有效筛选高危患者。
